# Features of the Defect Structure and Luminescence of Nominally Pure Lithium Niobate Crystals Produced Using Different Technologies

**DOI:** 10.3390/ma16010255

**Published:** 2022-12-27

**Authors:** Maxim Smirnov, Diana Manukovskaya, Nikolay Sidorov, Mikhail Palatnikov

**Affiliations:** Tananaev Institute of Chemistry—Subdivision of the Federal Research Centre “Kola Science Centre of the Russian Academy of Sciences” (ICT), 184209 Apatity, Russia

**Keywords:** lithium niobate, luminescence, defects, small radius polaron, band gap, luminescence center

## Abstract

We have established that luminescence in lithium niobate crystals both congruent and near-stoichiometric (R ≈ 1) is due to point defects in the cationic sublattice and intraconfigurational transitions in the oxygen-octahedral NbO_6_ clusters. We have also determined that the main contribution to the luminescence in the visible and near IR regions is made by luminescence centers with the participation of Nb_Li_ defects: the Nb_Li_-Nb_Nb_ bipolaron pair and the Nb_Li_-O defect in a congruent crystal. The minimum intensity of bipolaron luminescence has been observed in stoichiometric crystals obtained using different technologies. Weak luminescence of the Nb_Li_-Nb_Nb_ bipolaron pair indicates a small number of Nb_Li_ defects in the crystal structure. The number of Nb_Li_ defects in the crystal structure indicates a deviation of the crystal composition from stoichiometry.

## 1. Introduction

Every year, the requirements for the quality of materials for new applications in various industries are increasing. The development of new or the modification of existing technological approaches is required for their production and quality control [1,2]. Lithium niobate single crystal (LN, LiNbO_3_) is an important functional optical material widely used in telecommunications equipment, integrated optics and laser technology [2]. LN is a deeply defective oxygen-octahedral phase of variable composition with a wide homogeneity region on the phase diagram. This makes it possible to change the stoichiometry of the crystal (the Li/Nb ratio), as well as to introduce dopants into the structure, thereby changing the defect state and physical characteristics of the crystal [3,4,5]. 

A perfect stoichiometric crystal (SLN, Li/Nb = 1) has a transparency window in the region of 320–5000 nm [6]. However, real LiNbO_3_ crystals always contain point defects Nb_Li_ and trace amounts of impurities (~10^−3^–10^−4^ mol%). Nb_Li_ is niobium cations located in the lithium site. The concentration of such defects is especially high in crystals grown from a melt with an excess of niobium. (Li/Nb < 1). Thus, in a congruent crystal, the concentration of Nb_Li_ point defects is ≈6 at % [3]. Nb_Li_ defects necessitate maintaining the charge electrical neutrality of the crystal. This leads to the formation of a whole spectrum of point and complex defects in the crystal, which act as hole and electron capture traps. Practically significant physical properties of crystals depend on several factors. These properties include luminescence, the effect of photorefraction, the magnitude of spontaneous polarization and the strength of the coercive field. The factors influencing them are as follows: defects in the form of shallow and deep electron traps in the band gap; defects that change the polarizability of oxygen-octahedral clusters MeO_6_ (Me − Li^+^, Nb^5+^, dopant); ordering of the structural units of the cationic sublattice along the polar axis. Therefore, in order to create materials based on the LiNbO_3_ crystal for generating and converting laser radiation, in addition to reducing the photorefractive properties, it is necessary to reduce the number of defects in the crystal responsible for spontaneous radiative recombination in the optical region of the spectrum. 

Nominally pure nonlinear optical LiNbO_3_ crystals of congruent (R = Li/Nb = 0.946, CLN) and stoichiometric compositions differ significantly in photorefraction effect (optical damage) and coercive field strength, ~21 and ~5 kV/ mm, respectively [7]. SLN crystals grown from a melt with 58.6 mol% Li_2_O are characterized by a strong inhomogeneity of the refractive index along the growth axis [8]. In addition, such crystals are more sensitive to optical damage than CLN crystals due to the increased content of defects in them in the form of shallow electron traps [8]. CLN crystals with a low photorefraction effect can serve as efficient converters and modulators of laser radiation on periodically polarized micron and submicron domains. However, their application is limited by the high value of the coercive field [7]. Optical damage depends on the number of intrinsic defects. They form local energy levels of different depths in the band gap of the crystal. A relevant goal for the development of highly advanced optical materials with controlled (including the lowest possible) photorefraction effect is to establish the main electron relaxation processes, the nature of localized energy levels, and their energy distribution in the band gap of the LiNbO_3_ crystal. In this study, we have performed a complex analysis of the optical properties of LN crystals in the region of the intrinsic absorption edge, and also studied the photoluminescence (PL) spectra. Such spectra provide reliable information about the nature of luminescence centers and energy levels in the band gap [9,10,11,12]. This study establishes the distinctive features of photoluminescence, considers the influence of the composition and technology of nominally pure LN single crystals on the distribution of luminescence intensity in the optical region, and establishes the nature and localization of the most active luminescent centers in the crystal. It reveals a relationship between the optical characteristics of nominally pure LN crystals and their stoichiometry; the features of technologies for their production; the features of various luminescence centers localization in the crystal. 

## 2. Materials and Methods

The crystals were grown by the Czochralski in an air atmosphere on a Kristall 2 setup (Voroshilovgradsky zavod electronnogo mashinostroeniya, Voroshilovgrad, Russia) from a granular charge of LN synthesized at the ICT [13]. A congruent crystal (CLN) was grown from a congruent melt (R = 0.946). The first stoichiometric crystal (SLN) was grown from a melt with excess Li_2_O (58.6 mol%). The second stoichiometric crystal (SLN 6.0 wt% K_2_O) was grown using the HTTSSG technology from a congruent melt (R = 0.946 in a charge) with an alkaline flux K_2_O (≈6.0 wt%). The concentration of impurities traces in the crystals did not exceed 1 × 10^−3^ for Mo, Fe, Ti, Si, Pb, Ni, Cr, Co and 5 × 10^−4^ wt% for Al, Cu, Mn, V, Mg, Sn. All crystals were turned to a single-domain state by high-temperature electrodiffusion annealing during cooling at a rate of 20 °C/h in the temperature range from ~1240 to 890 °C under constant voltage [13]. The degree of single domain state was controlled by two methods: the value of the static piezoelectric modulus (d_333st_) of the crystal boule and the electroacoustic method. The latter is based on the analysis of the frequency dependence of the electrical impedance [13]. 

Studied samples were cut out in the form of rectangular parallelepipeds with dimensions ~8 × 7 × 6 mm^3^. Their edges coincided in direction with the crystallophysical axes X, Y, Z (Z is the polar axis of the crystal). The faces of the parallelepipeds have been polished carefully. 

The reflection (R(λ)) and transmission (T(λ)) spectra were recorded with a spectrophotometer SF-56 (Lomo-Microsystems Ltd., Saint-Petersburg, Russia). A deuterium lamp (190–340 nm) and a halogen lamp (340–800 nm) were used as radiation sources. The discretization step was 0.1 nm, the slit width was 6 nm. A photodiode was used as a photodetector. Photoluminescence spectra were recorded in 90-degree geometry from the volume of the studied samples using a spectrograph SOL SL-100 M (SOL instruments, Minsk, Belorus) with a CCD detector FLI ML 1107 Black Illuminated (Hamamatsu PHOTONICS K.K., Hamamatsu City, Japan) in the wavelength range of 380–800 and 800–1050 nm under normal conditions. A cw He-Cd laser (Kimmon KOHA, Fukushima, Japan, λ_ex_ = 325 nm, 15 mW) was used as a luminescence excitation source. The background signal was subtracted from each PL spectrum. 

The studied samples were heated in the range from 30 to 90 °C in steps of 5 °C at a rate of 4 °C/min to study the thermal quenching of PL. A body with a nichrome filament was used as a heater. An APS-7151 power supply (Aktakom (NPP “Eliks”), Moscow, Russia) was connected to the ends of the nichrome filament. The temperature was measured with a thermocouple of multimeter MY64 (Precision Mastech Enterprises Company Ltd., Hong Kong, China). 

In order to separate maxima from luminescent spectrum in thermal quenching experiment we constructed ln(I) dependence on the reciprocal temperature and approximate it using the Equation (1). This equation describes the temperature dependence of the bound exciton emission according to the Mott theory [14]:

(1) $$I\left(T\right)=I_{0}{\left[1+\overset{n}{\underset{i=1}{\sum }}c_{i}e^{\frac{E_{a}^{i}}{\mathrm{kT}}}\right]}^{-1}$$

where I_0_—radiation intensity at T→0, c_i_—normalization factor, $E_{a}^{i}—$ the activation energy at which thermal quenching of the emission occurs, k—Boltzmann constant (8.617 × 10^−5^ eV · K^−1^), T—temperature.

## 3. Results

### 3.1. Study of the Optical Homogeneity of Nominally Pure LiNbO_3_ Crystals in the Region of the Fundamental Absorption Edge

Nonlinear optical properties of LN crystal are determined by Li-O and Nb-O chemical bonds. The bonds also determine the geometry of MeO_6_ oxygen-octahedral clusters (Me − Li^+^, Nb^5+^, doping metal), and the clusters are the basis of the crystal structure. Traces of numerous cationic impurities (point defects), as well as more complex charged defects, such as OH hydroxyl groups, affect the structure of a nominally pure crystal. These defects redistribute charge in the crystal and change the lengths of Li-O and Nb-O chemical bonds of an ideal crystal structure. Variations in chemical bond lengths change the band gap, refractive index, optical absorption, and other parameters of the crystal.

**Figure 1:**
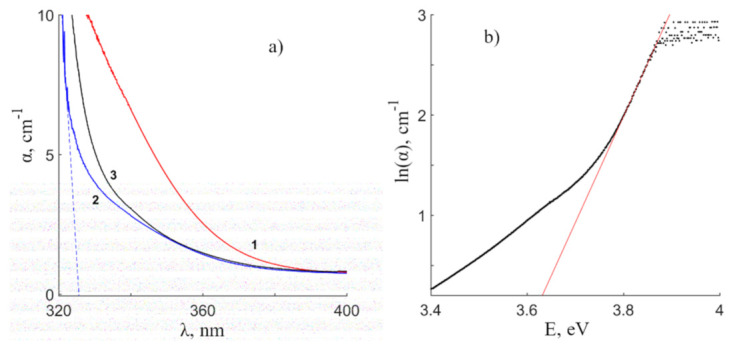
(**a**)—Optical absorption spectra of crystals: SLN (1), SLN (6.0 wt% K_2_O) (2), CLN (3). (**b**)—Dependence of ln(α) on E for calculating the Urbach energy of the CLN crystal.

Figure 1a shows the dependence of the absorption coefficient α(λ) on the excitation wavelength of CLN, SLN and SLN (6.0 wt% K_2_O) crystals. The spectra differ significantly from each other. Intense absorption is observed in the tail of the SLN spectrum from 330 to 380 nm, but it is absent from spectra of other crystals. This may indicate a large number of point defects in the form of small electron traps in SLN crystal. Point defects in the form of shallow electron traps create multiple shallow donor and acceptor energy levels near the valence and conduction bands in the band gap of the crystal. Such a crystal has a poorer optical quality in terms of shallow electron traps and a higher photorefraction effect, thus the larger opening angle of the PhotoInduced Light Scattering (PILS) indicatrix [15] than CLN and SLN (6.0 wt% K_2_O) crystals. 

The composition of a stoichiometric crystal is almost at the boundary of the homogeneity region [16]. This fact, together with the high volatility of lithium, causes a region of unstable solid solutions near the stoichiometric composition at temperatures close to the melting point and the Curie point. This makes it difficult to control growth conditions and obtain a highly perfect and optically uniform SLN crystal. The studied SLN crystal is not gyrotropic. However, the track of a laser beam propagating along the polar axis is intermittent, the modulation period is 0.33 mm [17]. The intermittent beam structure is observed mainly in the region of the focal waist and only for the SLN crystal. This fact indicates a high structural periodic inhomogeneity of the SLN crystal along the polar (growth) Z axis.

Figure 1a demonstrates that the absorption spectra of the CLN and SLN (6.0 wt% K_2_O) crystals are quite similar. However, SLN (6.0 wt% K_2_O) spectrum is shifted towards higher energies. The dashed line in Figure 1a indicates an approximation of the linear part of the SLN (6.0 wt% K_2_O) spectrum. The approximation was made in order to determine the fundamental absorption edge. The found values of the intrinsic absorption edge for the CLN, SLN and SLN (6.0 wt% K_2_O) crystals were 330.0, 326.9, and 326.0 nm, respectively. An increase in stoichiometry leads to a shift of the absorption edge towards shorter wavelengths, and the intrinsic absorption edge is maximal in the SLN (6.0 wt% K_2_O) crystal.

It is believed that excess Nb atoms in lithium sites (Nb_Li_ defect) contributes the most to the change in the position of the absorption edge in nominally pure LN crystals [18,19]. As is known, stoichiometric (R = 1) SLN and SLN (6.0 wt% K_2_O) crystals have a significantly smaller number of Nb_Li_ defects, while their amount in congruent (R = 0.946) CLN crystal should be maximal, approximately 6 wt% [3]. It is possible to qualitatively estimate the band gap and the R value based on the calculation of the concentration of lithium (c_Li_) from the position λ_abs_ at α ≥ 15 cm^−1^. According to the methodology proposed in work [18]:

(2) $$E_{g}=k\sqrt{50-c_{\mathrm{Li}}}+{\;E}_{0}$$

c_Li_ can be found as follows:

(3) $$c_{\mathrm{Li}}=50-{\left(\frac{E-E_{0}}{k}\right)}^{2}$$

where E_g_ is the energy corresponding to the absorption edge at the chosen value of α, c_Li_ is the concentration of Li_2_O in the crystal [mol%], k and E_0_ are constant coefficients. The parameter k is 0.189 ± 0.003 eV/mol%^1/2^; E_0_ is 4.112 ± 0.002 and 4.092 ± 0.002 eV for α = 20 and 15 cm^−1^ [18]. Since some crystals had different thicknesses, the calculation was made for LiNbO_3_ crystals satisfying the condition α ≥ 15 cm^−1^. The averaging of R value after calculation for α = 20 and 15 cm^−1^ is made.

Two models are used to describe the long-wavelength tail in the optical absorption spectrum: the model of the exponential shape of the absorption edge according to Urbach’s law and the model of direct and indirect interband transitions. To describe the spectral dependence of the absorption coefficient on the energy of the incident photon, the Urbach empirical law is used which is given by the following equation [20,21,22]:

(4) $$\alpha ={\;\alpha }_{0}e^{\frac{h\nu }{E_{U}}}$$

where α_0_ is a constant value, E_U_ is the Urbach energy which indicates the degree of disordering of the material. If we take the natural logarithm of both parts of Equation (4), then we can obtain a linear dependence of ln(α) on the energy of the incident radiation. The Urbach energy can be found from the slope of the straight line equation. Figure 1b shows the example of the Urbach energy determination by approximating the linear section to the abscissa axis of the dependence ln(α(E)) for a CLN crystal. The composition of the SLN (6.0 wt% K_2_O) crystal was not evaluated, since the large thickness of the sample does not allow us to obtain the corresponding values of λ_abs_ at α ≥ 15 cm^−1^. The obtained R values for CLN and SLN crystals are 0.948 and 0.952, Table 1. This confirms the literature data on a decrease in the concentration of Nb_Li_ point defects in the crystal lattice with an increase in stoichiometry [18,19].

Nb_Li_O_6_ complex defect centers change the shape (geometry) of oxygen-octahedral MeO_6_ clusters, chemical bond lengths, etc., and, accordingly, shift the fundamental absorption edge to the long-wavelength region. Crystals grown with different ratios of K_2_O in the melt show a gradual shift of the fundamental absorption edge towards shorter wavelengths depending on the K_2_O concentration, the maximum shift compared to CLN (Δλ_abs_ = 14 nm) corresponds to the SLN (10.5 wt% K_2_O) crystal [23]. In our case, the largest shift Δλ_abs_ = 4 nm is observed in the SLN (6.0 wt% K_2_O) crystal. This indicates that SLN (6.0 wt% K_2_O) crystal composition is very close to the ideal stoichiometric ratio, R = 1. The calculated Urbach energy (Table 1) confirms that this crystal has the highest compositional and optical homogeneity among studied samples. The exponential character of the absorption edge according to the Urbach model is usually in crystalline and amorphous materials [22]. The characteristic value of the Urbach energy for amorphous semiconductors is 10–100 meV [22]. It represents the width of localized states in the band gap of the crystal; localized states are associated with structural defects and disorder in the material [20,22]. This value can be used to estimate the disorder in the alternation of structural units of the cationic sublattice, the number of defects of different levels, the number of low-angle grain boundaries, etc., for crystals. The high value of the SLN Urbach energy is due to the shallow energy levels at the edges of the band gap of the crystal. In addition, the E_U_ value for a SLN crystal grown from a melt containing 58.6 mol % Li_2_O is comparable to that (96 meV) for a LiNbO_3_ crystal obtained using the VTE (vapor transport equilibration) method [22]. The E_U_ value of the CLN crystal is higher than that of other studied crystals. This can be explained by a large number of intrinsic point defects of the cationic sublattice (Nb_Li_, V_Li_, V_Nb_, etc.), and, therefore, by a higher degree of disorder of the main and impurity metal cations along the polar axis of the crystal.

**Figure 2:**
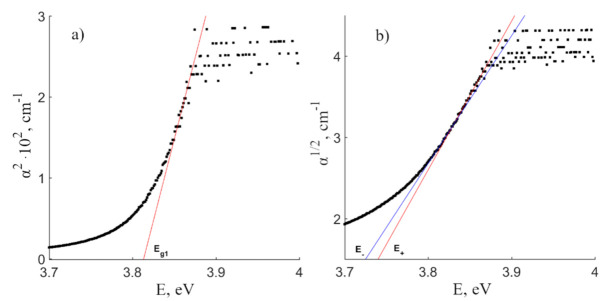
Dependencies α^2^(hν) and α^1/2^(hν) for determining direct (**a**) and indirect (**b**) transitions in CLN crystal.

The LN band gap is characteristic of a wide gap semiconductor [15,24]. The blurred absorption edge in LN—in addition to structural inhomogeneity—may indicate the indirect nature of interband transitions; the transitions are due to the electron-phonon interaction. Vibrations of the crystal lattice (phonons) can participate in the absorption near the edge of fundamental absorption, as well as on its “tail”. The influence of the electron-phonon interaction on the absorption spectrum can be estimated using the method proposed in works [22,24,25,26]. According to Tauc relation:

(5) $$\alpha \left(h\nu \right)\propto A{\left(h\nu -E_{g}\right)}^{n}$$

where A is a constant, hν is photon energy, E_g_ is allowed energy gap and n is an exponent, which depends on the nature of electronic transition in absorption process. For allowed direct and indirect transitions, n equals ½ and 2, correspondingly [24,25]. After mathematical operation, we obtain the following formulas:

$$\alpha^{2}=\mathrm{const}\left(h\nu -E_{g1}\right)\text{—for allowed direct transitions}$$

$$\alpha^{\frac{1}{2}}=\mathrm{const}\left(h\nu -E_{\pm }{}_{\;}\right)\text{—for allowed indirect transitions}$$

where E_g1_ is a band gap for the direct transition, $E_{\pm }=E_{g2}\pm E_{p}$, E_g2_—is a band gap for the indirect transition. Sign «+» corresponds to phonon absorption, sign «-» phonon emission and E_p_ is the phonon energy. Figure 2 shows the example of determining the band gaps for direct and indirect—with the participation of a phonon—transitions for a CLN. Table 1 presents the calculated values of studied crystals.

According to quantum mechanical calculations by the density functional theory (DFT method) performed in work [12], the electrons of the valence band are located in the 2p orbitals of O^2−^, and the 4d orbitals of Nb^5+^ form the conduction band. The photon should have the energy comparable to the band gap for charge transfer from the 2p orbital of O^2−^ to the 4d orbital of Nb^5+^. According to work [27], the top of the valence band is located at point Γ, and the bottom of the conduction band is shifted to the point 0.4 Γ—K, where the Γ and K points are the center of the Brillouin zone and one of the highly symmetric directions of the Brillouin zone in the LN hexagonal cell. A direct transition at the Γ point occurs initially during absorption. The indirect transition is accompanied by the absorption of crystal lattice phonons. The phonon spectrum of the first order of a LN crystal extends from 100 to 900 cm^−1^ (12–112 meV) [17]. The estimate of the direct and indirect transitions can be calculated by analogy with work [22]. Therefore, electron-phonon transitions which are determined by the laws of conservation of momentum and energy should be observed near the absorption edge. We have calculated the band gaps for direct transitions: 3.813, 3.846, and 3.826 eV (±0.004 eV) for CLN, SLN and SLN (6.0 wt% K_2_O) crystals. Stoichiometric samples have a larger direct transition E_g1_ value than a congruent crystal; this is due to the more perfect cationic sublattice of the SLN and SLN (6.0 wt% K_2_O) crystals. The order of alternation of cations and vacancies in these stoichiometric crystals is close to the order in an ideal crystal of stoichiometric composition (R = 1).

The values of the indirect transition (E_g2_) and phonons energies for these crystals are shown on Table 1. Phonons in the Table 1 were evaluated due to data in [17]. The excitation electrons most likely interact with E(TO) = 317–325 cm^−1^ or A_1_(TO) = 332–334 cm^−1^ phonons for the SLN (6.0 wt% K_2_O) crystal [17]. The A_1_(TO) phonon is a fundamental vibration of the crystal lattice; it is attributed to the “rigid” rotation of the O_6_ oxygen octahedron as a whole around the polar axis [17,28]. The closest vibrational frequency for CLN corresponds to the A_1_(TO) depolarized band at 120 cm^−1^. In turn, the band corresponds to the two-particle state of acoustic phonons with a zero total wave vector. This frequency belonging to the second-order spectrum is caused by a strong anharmonic interaction with the acoustic continuum of the lowest-frequency fundamental vibration A_1_(TO) of the symmetry type (quasi-soft mode) with the frequency of 254 cm^−1^ [17]. Its intensity depends on the stoichiometry of the sample: it is equal to zero in the Raman spectrum of a crystal of a strictly stoichiometric composition [17].

LiNbO_3_ crystals with a disordered cationic sublattice are characterized by a higher *E_p_* energy value. This may indicate a more complex character of electron-phonon interactions. A sufficiently developed second-order spectrum is characteristic of such crystals [29]. Quasiparticles can be formed from interacting phonons or mixing of one-phonon states with two-phonon states due to the Fermi resonance. The calculation by the method of direct and indirect transitions is quite sensitive to changes in the approximation parameters of the linear section, therefore, a strong error can be introduced into the measurements. On the other hand, the model of direct and indirect transitions poorly describes the change in the absorption edge of SLN.

The long-wavelength “tail” in the absorption curve can also be due to trace amounts (≈10^−3^–10^−4^ wt%) of uncontrolled impurities. They enter the crystal during growth from the melt. Thus, the tail might be not related to fundamental optical transitions. Localized shallow energy levels near the top (bottom) of the valence band (conduction band) can act as impurities. The absorption of some impurities, especially Fe, is near the absorption region of the LN crystal. This can also lead to broadening of the long-wavelength absorption shoulder in stoichiometric LN crystals ([Li_2_O] = 49.9 mol%) [18]. The variance in the values of E_g1_ and E_g2_ presented in our paper and work [22] is because of different thicknesses of samples.

An SLN crystal (R = 0.952) has a significantly less developed defect structure than CLN (R = 0.948) and, accordingly, a lower photorefractive sensitivity and a coercive field. However, study of the edge of optical absorption has revealed that SLN has worse optical quality than CLN and SLN (6.0 wt% K_2_O). This can be explained as follows: obtaining large-sized commercial SLN with high compositional and optical uniformity is extremely difficult. SLN crystals can be grown from melts with an excess of the alkaline component ([Li_2_O] ≈ 58.6 mol%). Growing crystals of high optical quality from such melts is a very complex technological problem. Crystallization parameters are difficult to control because of the deviation of the melt composition from congruent. The instability is caused by a discrepancy between the composition of the crystal and the composition of the melt and, as a consequence, changes in the composition of the melt during crystal growth. A significant difference in the compositions of the melt and crystal necessitates a decrease in the growth rate (<0.1 mm/h) compared to congruent crystals. Congruent crystals are usually grown at a growth rate of ~3–5 mm/h [9]. A decrease suppresses concentration supercooling, which leads to noticeable changes in the composition of the crystal at various stages of growth. However, even in this case, a crystal of stoichiometric composition with a diameter of only ~15 mm and a length of only 10 mm can be grown from 1000 g of the melt. The homogeneity of the refractive index of such crystals Δn is comparable to that of congruent crystals (Δn~2 − 5 × 10^5^ cm^−1^). Thus, growing optically homogeneous stoichiometric LN crystals of sufficiently large sizes is difficult. Significant inhomogeneity of the composition along the length of the crystal boule arises during growth and leads to a large scatter of physical parameters over the volume of the crystal.

A near stoichiometric crystal (NSLN) can be grown from a melt of a congruent composition with the addition of an excess of another alkaline component to the melt: ~6 wt% K_2_O. Growing such a crystal is a typical top seeded solution growth. The K_2_O solvent is quite unique; it allows one to obtain sufficiently large NSLN crystals comparable in homogeneity with congruent crystals. This is how the SLN (6.0 wt% K_2_O) crystal studied in this work was grown. At the same time, potassium is absent in the SLN crystal (6.0 wt% = 9.3 mol% K_2_O). The ionic radii of Li^+^ and K^+^ are 0.68 Å and 1.38 Å, respectively. With such a significant (more than twofold) difference in ionic radii, isomorphic substitution of related elements of the alkaline group (lithium by potassium) in the cationic sublattice of the crystal seems unlikely. Therefore, the composition of the melt during crystal growth changes much less than when growing an SLN crystal from a melt with a significant excess of Li_2_O ([Li_2_O] ≈ 58.6 mol %). Thus, the compositional and optical homogeneity increases in the series of SLN, SLN (6.0 wt% K_2_O), and CLN crystals.

### 3.2. PL of CLN, SLN, SLN (6.0 wt% K_2_O) Crystals in the Visible and Near-Infrared Spectrum

An endless crystal lattice can have such defects as Frenkel defects, interstitial and substitutional chemical impurities. The actual crystal size is limited; at the interface between two media (crystal-environment) its physical properties undergo a sharp jump. According to the ideal crystal growth theory [30], the “crystal-environment” interface is an atomically smooth surface. Real crystals always have numerous near-surface defects, in particular, Schottky defects, adsorbed atoms and molecules, screw dislocations, and macrodefects. The surface of crystals grown in an air atmosphere can adsorb water vapor molecules, thereby creating multiple hydrogen bonds above the crystal surface [31]. In this case, the parameters of elastic interaction in the boundary layer change [32]. Micro- and macro-defects, chips, cracks, grain boundaries, domain walls can act as surface luminescence centers; the defects significantly change the properties of the near-surface crystal layer in the relative to its volume.

Thus, the PL of LN crystals can be divided into two components. The first is based on the excitation of PL in surface defects (chips, cracks, grain boundaries, roughness) of a LN crystal; the spectral distribution of this part is observed in the blue region of the spectrum. The second is a «bulk» or «volume» luminescence. It is possible that the definition of “bulk photoluminescence” is not entirely correct due to the low ability of UV laser radiation to penetrate deep into the crystal. However, in order to avoid misunderstandings, in this paper by volume PL we will understand luminescence that is spectrally different from the surface one. Volume luminescence is concentrated in the visible and near-IR regions [33,34,35]. Due to hardware limitations, PL in the visible and near-IR regions will be considered separately in this paper. Surface PL, due to the abundance of defects, is 5 × 10^2^ times more intense than volume PL. Moreover, the latter strongly depends on the stoichiometry, dopant concentration, and a number of other features of the crystal.

#### 3.2.1. Surface PL in CLN, SLN, SLN (6.0 wt% K_2_O) Crystals

**Figure 3:**
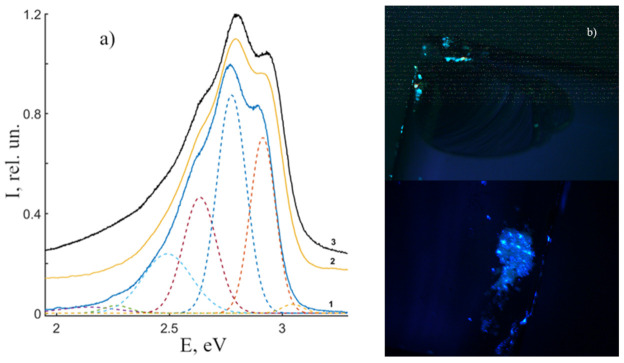
Surface PL spectra of nominally pure LN crystals (**a**): (1)—CLN, (2)—SLN, (3)—SLN (6.0 wt% K_2_O). Microimages of PL on the surface of the CLN crystal (**b**).

Let us consider the surface luminescence of nominally pure LN crystals and then proceed to the study of radiative recombination of luminescence centers in their bulk. A number of works [34,36] have already reported this luminescence. Figure 3a demonstrates normalized PL spectra of CLN, SLN, SLN (6.0 wt% K_2_O) crystals. Figure 3b also demonstrates an image of excited luminescence of the near-surface layer of the CLN crystal. The figure reveals that PL spectra of studied crystals are a wide luminescence band of complex shape with the intensity maximum at E_max_ = 2.75 eV. We have decomposed the spectra into a series of individual maxima of the Gaussian shape, Figure 3a. Table 2 demonstrates characteristics of separate peaks: I (rel. un.)—intensity, E (eV)—position, ΔE (eV)—full width at half maximum (FWHM). Additional maxima are observed at ~2.6 and 2.9 eV; other maxima are located in a low-energy luminescence “tail”.

The main bands (3–6, Table 2) of SLN and SLN (6.0 wt% K_2_O) are shifted to the high-energy area relative to the same bands of CLN: ΔE_3_, ΔE_4_ ≈ 0.03, ΔE_5_ ≈ 0.02–0.03, ΔE_6_ ≈ 0.03–0.04 eV. This fact confirms data on fundamental edge shift (Figure 1a) and thus positions of localized energy levels in the band gap of crystals. A shift to the high-energy area indicates a better ordered cation sublattice and a more perfect shape of oxygen octahedra in the surface area of stoichiometric crystals SLN and SLN (6.0 wt% K_2_O).

Spectra of studied crystals are obviously similar (Figure 3a); we will consider a band’s origin to be connected with the same luminescence centers. The intense maximum at 2.78 eV is due to radiative recombination in the Nb_Nb_^4+^-O^−^ pair of main niobium octahedron [37]. It suggests that the 2.97 eV band can be attributed to Zn dopant in the LN crystal [34]. In our case, this band has a maximum at 2.91 eV. Due to our data, even pure crystals not doped with Zn have the 2.91 eV PL emission band (Figure 3a). The reason for appearance of two near PL bands at 2.91 and 2.78 eV can be the following. Two different Nb-O distances along the polar axis exist in the main niobium octahedron of the LN crystal. The short distance is 1.879 Å, it forms a covalent bond; the long bond is 2.126 Å, it has an electrostatic character [3]. Therefore, the emission band at 2.91 eV in the PL spectra of the studied crystals can be attributed to radiative recombination of the other Nb_Nb_^4+^-O^−^ pair in the main niobium octahedron. The difference in the relative intensities of these two luminescence bands is minimal for SLN and SLN (6.0 wt% K_2_O) crystals (ΔI_5–6_ = 0.009 and 0.028, respectively), Table 2. This indicates a more uniform distribution of the absorbed energy between two Nb_Nb_^4+^-O^−^ pairs in SLN crystals relative to the CLN crystal. This feature may be due to the more regular shape of oxygen octahedra in crystals with the composition close to the stoichiometric.

On the other hand, the niobium ion in the LN crystal structure is surrounded by six oxygen ions. Due to the crystal field theory, in this case, 4d orbitals of Nb are released of degeneracy and splitting. This means that d_x2-y2_, d_z2_, d_xy_, d_yz_, d_xz_ energy orbitals are no longer equivalent to each other. The magnitude of splitting by the ligand field depends on the type of ligand, the coordination number of the central atom, and the distance between atoms [38]. In LN crystals, the magnitude of splitting depends on the localization of the Nb^5+^ ion in the cationic sublattice. Moreover, a further shift of the central ion position in the oxygen octahedron leads to overlapping of shells (atomic orbitals) of niobium and the ligands with the formation of molecular orbitals in an ideal NbO_6_ octahedron. In a LN crystal, the O^2−^ 2p orbitals are completely filled with electrons and form the valence band, while the Nb^5+^ 4d orbitals form the conduction band [12,39]. Excitation is accompanied by charge transfer from the 2p orbital of O^2−^ to one of the 4d orbitals of Nb^5+^, which corresponds to an increase in the electron charge density of Nb^5+^ along the octahedral bonds. During electronic relaxations, first, nonradiative transitions to the smallest excited state of Nb^4+^ occur, followed by recombination with a hole in the 2p orbital of oxygen. The oxygen octahedrons are distorted in a LN crystal; the symmetry of the oxygen octahedron in an ideal SLN crystal is far from cubic (O_h_). In addition, the Nb^5+^ ions are displaced from the centrosymmetric position in the MeO_6_ oxygen-octahedral cluster; this fact causes the spontaneous polarization and the ferroelectric properties of the crystal. All this should lead to an additional removal of the degeneracy of the energy levels of the NbO_6_ group and to a nonzero probability of radiative recombination from them. In addition, the “crystal-environment” interface can also introduce corrections into the arrangement of the energy levels of the NbO_6_ group due, for example, to adsorbed oxygen, CO_2_, etc., molecules on the surface of the crystal. Thus, the nature of the most intensive bands can be associated with intraconfigurational transitions in the oxygen-octahedral NbO_6_ group.

The discussion proves that the luminescence in the blue region hardly depends on the crystal composition. The spectrum of such blue LN PL reminds only of radiative recombination in ceramic solid solutions of niobates-tantalates of alkali or REE [40]. However, in such materials, the coordination number for Nb^5+^ is usually four, not six, as in the LN crystal. This leads to a different system of energy levels; 4d orbitals of Nb split in a tetrahedral field. 

In the low-energy region (E < 2.5 eV), luminescence centers are mainly due to intrinsic defects in the crystal lattice. In the CLN crystal, one of these defects is a niobium in the lithium site, Nb_Li_. The concentration of such defects in a congruent crystal reaches ~6 at% [3]. Radiative recombination of such a defect is near 525 nm (2.36 eV) [10]. As R nears 1, the amount of such defects decreases. Nb_Li_ defects are absent from a strictly stoichiometric crystal. However, relative intensity of this band in SLN PL spectrum is five times higher than that in CLN PL spectrum. This indicates additional impurity luminescent centers in studied SLN and SLN (6.0 wt% K_2_O) crystals. Such impurity defects should have a large absorption cross section and efficient radiative recombination. They exist because isomorphic insertion in lithium sited dominates over insertion in niobium sites. Impurities and niobium predominantly occupy lithium sites because lithium octahedron is larger than the niobium one [3]. Even very small amounts of Nb_Li_ defects in SLN and SLN (6.0 wt% K_2_O) crystals can provide enough lithium octahedrons to be occupied by an impurity. However, it is impossible to determine which impurity contributes to the luminescent signal of a nominally LN pure crystal: their concentrations are very low, at trace amounts (10^−4^ wt%).

According to the electroneutrality mechanism, niobium in the lithium site creates either a niobium vacancy (4/5V_Nb_) or four lithium vacancies (V_Li_) [3,41,42]. According to cathodoluminescence studies, radiative recombination at 2.5 and 3.3 eV can be caused by lithium and niobium vacancies on the crystal surface, respectively, [43]. If we assume (in the first approximation) that the relative intensity of the luminescence band corresponds to the number of luminescence centers and proceed from the model of lithium vacancies, then, using the data in Table 2, we can calculate the approximate deviation from this model for each studied crystal. The calculated deviations are Δ_Li SLN_ = +0.44, Δ_Li CLN_ = −0.64, Δ_Li SLN K2O_ = +0.66. The sign of «+» or «-» corresponds to an excess or a deficiency of V_Li_. The value indirectly indicates the presence of additional multiply charged elements in lithium sites on the surface of SLN and SLN (6.0 wt% K_2_O) crystals. Additional slight luminescence is observed in SLN at 3.3 eV (Figure 3a). This indicates the increase in the niobium vacancies luminescence centers. Thus, according to the model of lithium vacancies, both V_Li_ and V_Nb_ defects are present in the near-surface layer of the CLN crystal. Additional impurity ions are localized in main lithium sites of the SLN crystal; these impurities increase the excess of V_Li_ defects.

#### 3.2.2. Volume PL in CLN, SLN, SLN (6.0 wt% K_2_O) Crystals in the Visible Region

**Figure 4:**
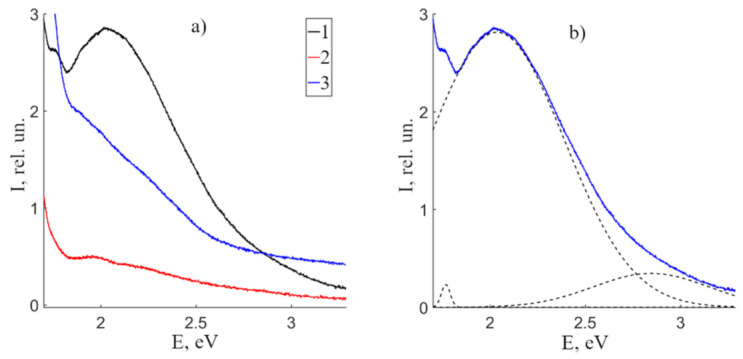
Volume PL spectra of nominally pure CLN (1), SLN (2) and SLN (6.0 wt% K_2_O) (3) crystals (**a**). An example of the of the CLN PL spectrum decomposition into a series of individual maxima (**b**).

Let us proceed to the volume PL of CLN, SLN and SLN (6.0 wt% K_2_O) crystals in the visible and near-IR regions. Figure 4 demonstrates the PL spectra of the studied crystals. CLN spectrum is a wide luminescent halo with a maximum at 2.04 eV. An increase in the crystal stoichiometry strongly quenches the luminescence, the integrated intensity of the SLN and SLN (6.0 wt% K_2_O) crystals spectra is 80% and 30% less than for CLN. In addition, the luminescence intensity increases in the low-energy region of the spectrum (<1.8 eV). The sharpest increase is in SLN (6.0 wt% K_2_O) crystal. The luminescence “tail” in the near-IR region of these crystals will be discussed separately.

Based on our experimental data on the temperature quenching of luminescence (see the Section 3.2.3) and data from papers [10,37,44,45], we will decompose on the basis of luminescence bands, the intensity of which decreases with increasing crystal temperature. Table 3 presents the characteristics of the spectra of the studied crystals after decomposition into a number of individual Gaussian maxima. The maximum near ~1.91 eV may be attributed to the generation of multiple harmonics of laser radiation exciting the PL spectra, it will not be discussed in this paper.

The perfect stoichiometric (R = 1) LN crystal is free of Nb_Li_ defects, thus it can have only one luminescent center. It is caused by Nb^5+^ cation linked with an O^2−^ oxygen anion with electrostatic or covalent bonds. Such niobium ion is located on its site in O_6_ octahedron. An electron transfers to the conduction band when excited, after that it is captured and a small radii polaron Nb_Nb_^4+^ forms (the so-called «free small polaron» [46]). The polaron is stabilized by lattice distortions. A hole is formed in the valence band, which is localized in the 2p orbital of O^−^ («free hole polaron» [46]). Next, the electron in the 4d orbital of Nb^4+^ recombines the hole in the 2p orbital of O^−^. The radiation of this recombination lies in the blue-green area, due to literature [44] and our data on surface luminescence. A violation of stoichiometry (R < 1) in LN single crystals leads to a lithium deficiency. Excess niobium atoms locate in lithium sites due to density measurements and XRD data. This forms Nb_Li_ defect in lithium niobate crystal [3]. According to the band theory of solids, the Nb_Li_ defect forms a localized energy level in the band gap of the crystal. The defect can localize one conduction band electron in the 4d^0^ orbital, which forms the second small radii polaron Nb_Li_^4+^ (4d^1^), «bound small polaron» [44,46]. The radiative recombination of Nb_Li_^4+^-O^−^ centers is attributed to the near-IR region [35].

**Figure 5:**
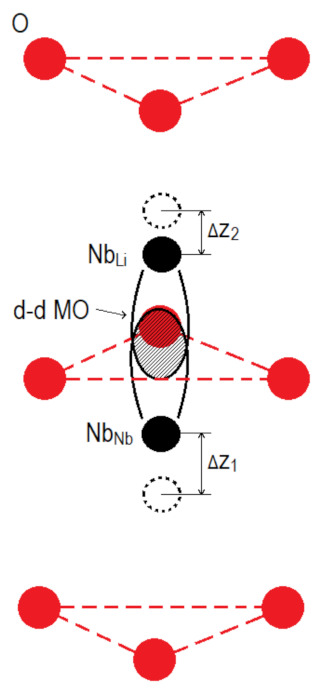
Formation of a bipolaron pair Nb_Li_-Nb_Nb_ in the structure of a LN crystal.

**Figure 6:**
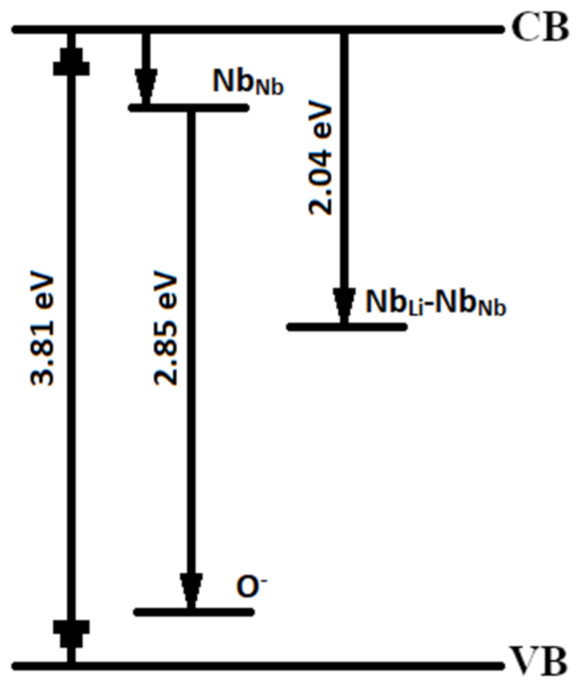
Qualitative scheme of the main channels of radiative recombination in the visible region of the spectrum according to the data of the fundamental absorption edge and PL of the CLN crystal.

Thus, CLN crystals have two main luminescence centers: Nb^5+^ cations in their own sites in O_6_ octahedra and Nb^5+^ cations in Li^+^ sites (a Nb_Li_ point defect) [3,10,44]. Papers [10,44] demonstrate that luminescence of Nb_Li_ defects is at 510–520 nm (2.39–2.43 eV) and is predominantly activated in real nominally pure LN crystals at λ_excit_ ≥ 300 nm. The maximum at 2.00 eV in the PL spectra of the crystals under study (Figure 4) is due, in our opinion, to the formation of Nb_Li_-Nb_Nb_ bipolaron. This can be explained as follows. Initially, an electron in the 4d^1^ orbital of Nb_Li_ must change electrostatic interactions with neighboring ions. According to the EPR data, the electron is stably localized in the d_z2_ orbital, the orbital is co-directed with the third-order axis (polar axis) of LN crystal [47]. In this case, the Coulomb repulsion between the Nb_Nb_ and Nb_Li_ defects decreases, the Nb_Li_^4+^ defect is shifted by Δz towards the nearest Nb_Nb_^5+^ cation. A common molecular orbital Nb_Nb_-Nb_Li_ is formed from atomic orbitals [47]. The expansion of the oxygen triangle between the pair accompanies the formation of a molecular orbital. The electron density is concentrated closer to the Nb_Li_ defect [48]. The charge carrier can be captured from the conduction band due to the formation of the Nb_Li_-Nb_Nb_ (4d^1^–4d^0^) pair, Figure 5. As a result, emission occurs in the visible region with the formation of a bipolaron pair Nb_Li_^4+^-Nb_Nb_^4+^ (4d^1^–4d^1^). Optical transitions of a singlet Heitler-London bipolaron Nb_Li_^4+^-Nb_Nb_^4+^ pair were obtained according to quantum mechanical calculations [45], the values are 2.0 and 2.3 eV, respectively. Moreover, the bipolaron Nb_Li_^4+^-Nb_Nb_^4+^ (4d^1^–4d^1^) pair must be a deeper electron trap and have a lower energy than Nb_Li_, because crystal lattice distortions stabilize the pair and ions of different valences interact electrostatically. The formation of a bipolaron pair is the capture of a photoexcited electron in the conduction band accompanied by emission in the region of ~2.00 eV. Apparently, there are two competing processes: the formation of a bipolaron pair and the recombination of an Nb_Li_^4+^ electron with a free hole polaron O^−^. The formation of a bipolaron pair depends on the degree of overlap of the d-d orbitals of neighboring Nb, that is, on the distance between Nb_Nb_ and Nb_Li_ in the crystal lattice. A qualitative diagram of the energy levels of these luminescence centers is shown in Figure 6.

PL maxima at 2.04, 2.06 and 2.04 eV observed in CLN, SLN and SLN (6.0 wt% K_2_O) spectra are caused by Nb_Li_^4+^-Nb_Nb_^4+^ bipolaron. The amount of Nb_Li_^4+^-Nb_Nb_^4+^ bipolarons should decrease with an increase in R; thus, intensity of PL maxima attributed to the bipolaron should also decrease. This is why the amount of Nb_Li_^4+^-Nb_Nb_^4+^ complex defects and luminescence centers increases as the crystal composition gets farther from stoichiometry: it is maximal in CLN and minimal in SLN, Table 3. In addition, the ratio Li/Nb = 0.952 (Table 1) in CLN crystal also indicate maximal luminescence in the visible region compared to the luminescence of SLN. Moreover, the distance between adjacent atoms Nb_Nb_ and Nb_Li_ is probably larger in SLN than in CLN crystals. This can lead to a weaker overlap of their d-d orbitals. As a result, the probability of luminescence in a given spectral region should additionally decrease. Our study reveals presence of Nb_Li_^4+^-Nb_Nb_^4+^ luminescence centers in SLN (6.0 wt% K_2_O) crystal despite the fact that its composition should be near-stoichiometric (R = 1). This means that Nb is not completely substituted in lithium sites in the SLN (6.0 wt% K_2_O) crystal. Indeed, Nb_Li_^4+^ defects occur in SLN (6.0 wt% K_2_O) crystal according to X-ray studies [49]. 

The band at 2.85–2.90 eV is attributed to the radiative recombination of Nb^4+^-O^−^, considering that niobium occupies its own octahedron [44,50]. The capture of electron by Nb^4+^ (small radii polaron or free small polaron) is accompanied by splitting of the EPR spectrum and the shift of g in a distorted octahedral cluster [51]. The capture of the O^-^ hole is accompanied by a broad EPR signal around g = 2, which is characteristic of many oxide materials [51]. In addition, the 2.85 eV maximum is comparable to the maximum in the surface PL spectrum of nominally pure CLN, SLN and SLN (6.0 wt% K_2_O) crystals. A similar situation is also characteristic of ceramic solid solutions of alkali and REE niobates [40].

Maxima 3 and 4 are shifted to the high-energy area in PL spectra of SLN and SLN (6.0 wt% K_2_O) compared to the maxima in CLN crystal, Figure 4, Table 3. The shift can be associated with a change in the optical absorption of crystals in the region of the fundamental absorption edge and, accordingly, with a change in the band gap. The previous section has revealed the fundamental absorption edge in SLN and CLN crystals, 326.9 and 330.0 nm (3.793 and 3.757 eV). The difference in band gaps is 0.036 eV. The energy state of the levels in the band gap of the SLN (6.0 wt% K_2_O) crystal is intermediate between them. The shift may be due to a change in the polarizability that appears when the composition of the crystal and, thus, oxygen-octahedral clusters MeO_6_ changes. The clusters in their turn determine optically nonlinear and ferroelectric properties of LN crystal.

We would like to draw attention to the fact that volume PL is two orders of magnitude weaker that surface PL. This may be due to the small absorption and emission cross sections of defect centers involved in electronic relaxations. In addition, the concentration of point and impurity defects, screw dislocations, Schottky defects, etc., is maximal in the area of the surface defects. The intensity of radiative recombination in the visible region is low due to the scattering of most of the absorbed energy on vibrations of the crystal lattice. Recombination in the visible region happens on Nb_Li_ defect centers. According to [3], the amplitude of thermal vibrations of the Li^+^ ion located in the lithium octahedron is approximately seven times greater than the vibration amplitude of the Nb^5+^ ion located in its own octahedron. The absorption of atoms and molecules of the environment on the LN surface can introduce an additional electrostatic effect, which will change the channels of radiative and nonradiative transitions. Moreover, an increase in the excitation radiation time, including the UV region, transfers energy to the near IR region [51]. In our case, the PL spectra are stationary, the intensity remains constant in time. This indicates saturation and establishment of thermodynamic equilibrium between generation, transfer and recombination. The amount of Nb_Li_ defects in studied crystals is quite low, thus volume PL is weaker than surface one. Regular electron-hole Nb_Nb_^4+^-O^−^ pairs of LN crystal participate in surface PL. 

#### 3.2.3. Volume PL in CLN, SLN, SLN (6.0 wt% K_2_O) Crystals in the Near-IR Region

**Figure 7:**
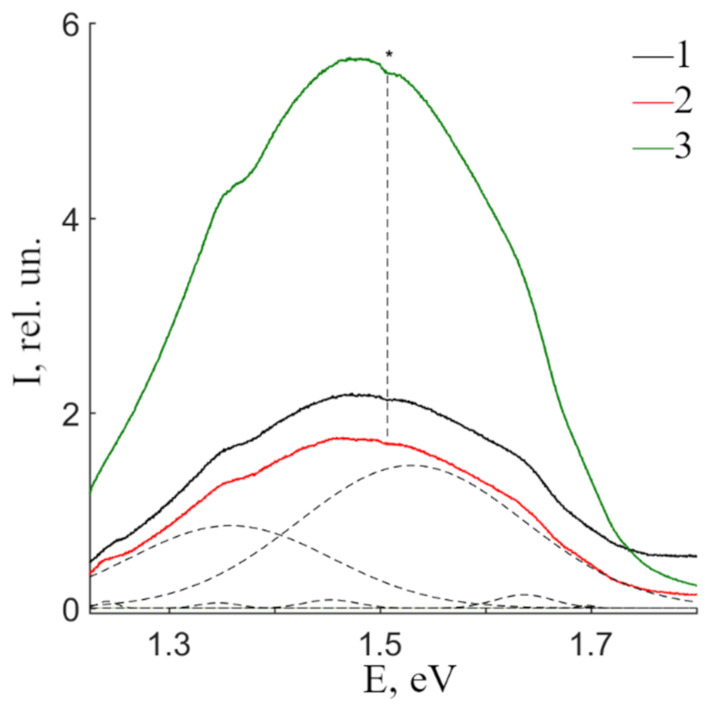
PL spectra of CLN (1), SLN (2) and SLN (6.0 wt% K_2_O) (3) crystals in the near-IR region. * denotes a dip at 1.5 eV in all presented spectra.

Figure 7 demonstrates PL spectra of CLN, SLN and SLN (6.0 wt% K_2_O) crystals in the near-IR region. Spectra of all three studied crystals is a complex asymmetric luminescence halo with a maximum at 1.48 eV. Integral intensity of SLN is 24% smaller than that of CLN, at the same time, integral intensity of SLN (6.0 wt% K_2_O) is 2.5 times greater. This indicates a strong dependence of PL in the near-IR region on LN crystals technology. The dependence of luminescence intensity on the crystal technology is clearly demonstrated in [35]. The SLN (6.0 wt% K_2_O) crystal was grown due to HTTSSG technology [35]. Its spectrum has two separate maxima at 1.6 and 1.4 eV [35]. On the contrary, a wide luminescent band is observed in CLN spectrum [35]. The intensity in the spectrum probably depends on the wavelength of the excitation radiation, since the excitation of the PL spectrum by visible laser radiation dissociates the bipolaron pair into individual polarons of small radius, followed by their recombination at hole centers with emission in the near-IR region [35].

A dip at 1.5 eV is present in all CLN, SLN and SLN (6.0 wt% K_2_O) crystal spectra, asterisk on Figure 7. Apparently, it is connected with reabsorption: the emission spectrum overlaps with the absorption spectrum of some luminescence center. The dip depth is small and weakly depends on the composition. Thus, we assume this center to be caused by trace amounts of uncontrolled impurity in the studied crystals. Figure 7 clearly demonstrates that the halo is complex, and individual components can be identified. Table 4 presents spectral characteristics of bands separated as Gaussians.

Separation reveals at least seven individual luminescence maxima at 1.24, 1.35, 1.36, 1.46, 1.54, 1.64 and 1.70 eV. Two maxima at 1.36 and 1.54 eV dominate over others in PL spectra of all studied samples in the near-IR region. Other bands are low-intense emission peaks, Table 4. The main maximum is at 1.54 eV. Its intensity is 1.3 times smaller in SLN than in CLN, and 2.3 times higher in SLN (6.0 wt% K_2_O) than in CLN. The ratio I_3_/I_5_ is 0.51, 0.58 and 0.79 in CLN, SLN and SLN (6.0 wt% K_2_O) crystals, respectively. Thus, a change in the crystal composition causes redistribution in luminescence intensity of maxima at 1.54 and 1.36 eV; the intensity of the 1.36 eV maximum increases with an increase in the LN crystal stoichiometry.

Additional low-intense maxima at 1.24, 1.35, 1.46, 1.64 and 1.70 eV might be caused by luminescence impurities, such as Cr^3+^, Mn^3+^ etc. For example, 1.70 eV band corresponds to traces of Cr [52]. On the other hand, the analysis of gradient-activated crystals showed [52]: despite the main R-bands of Cr^3+^ ions, spectra contain a wide-band emission at electronic-vibrational ^4^T_2_–^4^A_2_ transitions in the near-IR region. The Cr position of in the crystal cationic sublattice strongly affects the luminescence: Cr in the lithium site gives luminescence at 1.4 eV, and in niobium site—at 1.3 eV [52].

Due to data from the literature, the nature of the near-IR radiation is associated with the polaron luminescence of lithium niobate [35,46]. Publications devoted to the study of polaron luminescence are rare. In particular, the most studied polarons of small radius in lithium niobate are Nb_Li_ and Nb_Nb_; their emission is presented in [53,54]. The number of small-radius polarons Nb_Li_ and Nb_Nb_ can be controlled using thermal or optical dissociation of the Nb_Li_-Nb_Nb_ bipolaron pair [50,54]. The recombination of an electron in the 4d orbital of Nb^4+^ with a hole in the 2p orbital of O^−^ gives luminescence in the blue region, as described above (Section 3.2.1). The Nb_Li_ point defect is a deep electron trap, which indicates location of the local energy level closer to the middle of the band gap of the crystal [48,55]. Therefore, the electron-hole recombination of Nb_Li_-O seems to be possible in the near-IR region. Consequently, only one emission band should appear in the spectrum. However, the presence of two intense luminescence bands at 1.54 and 1.36 eV indicates a more complex nature of the radiative recombination channels. These emission bands are apparently associated with the Nb_Li_ defect and depend on the technology of growing LN single crystals. The intensities of 1.54 and 1.36 eV SLN bands are 22 and 11% lower than the intensities of the CLN bands. This indicates a decrease in the number of niobium atoms in lithium sites in the SLN crystal. Moreover, a decrease in the number of Nb_Li_ defects is accompanied by a decrease in defects that compensate for excess |4e| charge of Nb_Li_. Lithium and niobium vacancies (V_Li_ and V_Nb_) act as such defects [3,41,42].

Further considerations about intrinsic defects and their effect on luminescent properties will be described in terms of the lithium vacancy model [41] since this model dominates in describing the arrangement of cations in a LN crystal of various composition and genesis. The composition of the SLN (6.0 wt% K_2_O) crystal (R = 0.99) is close to the ideal stoichiometric composition (R = 1) [56]. Nevertheless, intense polaron luminescence is observed in the crystal. Additionally, a PILS indicatrix opens in this crystal during 60 s under action of a 160 mW laser radiation [57]. This indicates the presence of Nb_Li_ defects and a number of other donor and acceptor capture traps (shallow electron traps) in SLN (6.0 wt% K_2_O) crystal. The defects increase photorefractive effect [57]. Anyway, we can claim that both SLN (6.0 wt% K_2_O) and SLN crystals have a greater amount of shallow electron traps, than CLN crystal. Shallow electron traps can also participate in recombination processes.

**Figure 8:**
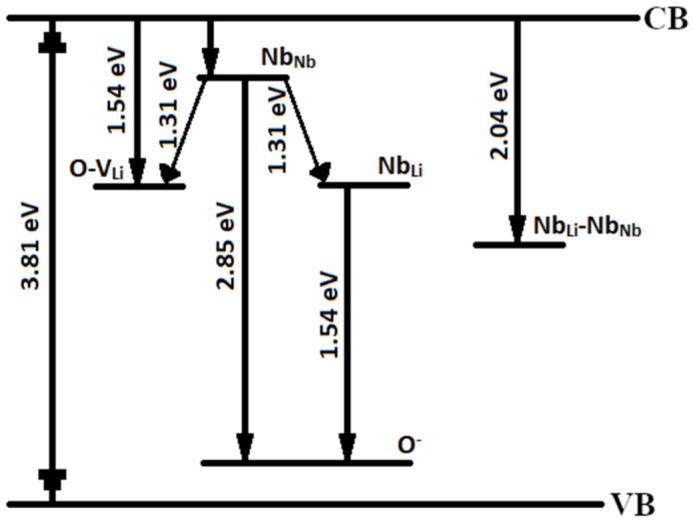
An enhanced scheme of the main channels of radiative recombination in the visible region according to the fundamental absorption edge and PL of CLN crystal.

Authors in [54] attribute 920 and 854 nm bands in LN spectrum to the following luminescent centers. The first band is attributed to radiative recombination of a free electron of the conduction band with a hole polaron O^−^. The second band can be caused by two reasons: intrinsic Nb_Li_ defects or formation of polaron Nb_Li_^4+^ when an electron is captured on Nb_Li_^5+^, the charge than is transferred to the levels of Nb_Nb_^4+^ [54]. Note that Nb_Li_ defects amount depend on the amount of bipolaron pairs in the crystal [11]. Upon photoexcitation near the fundamental absorption edge, a free hole is formed in the valence band; the hole is localized to the 2p orbital O^−^. The coordination number of oxygen is four, two niobium and two lithium atoms are surrounding an oxygen atom in an ideal stoichiometric crystal [58]. Since the Nb_Li_ defect leads to the formation of lithium vacancies, therefore, a V_Li_ defect can appear in the coordination environment of oxygen, together with which oxygen forms small hole traps O–V_Li_. These levels are located in a band gap; their energy should be higher than the energy of O^−^ [46]. Consequently, the recombination of electron at Nb_Nb_^4+^ with a hole polaron O^−^ leads to luminescence in the visible range, Figure 8. Possibly, electron-hole recombination occurs between the electron of the conduction band and the O^−^-V_Li_ centers with emission in the near-IR region, Figure 8.

On the other hand, Nb_Li_ defects act as deep electron traps, thus electron-hole recombination Nb_Li_-O should manifest in the near-IR region. This transition can also be attributed to 1.54 eV band, Table 4. The visible band at 2.85 eV is attributed to Nb_Nb_-O luminescence centers. At this, the energy difference is ΔE = 2.85–1.54 = 1.31 eV, this correlates well with the emission at 1.36 eV, Table 4. We can assume that charge transfer between Nb_Nb_ (4d^1^) and Nb_Li_ (4d^0^) is accompanied with the emission at lower energies [35]. Figure 8 presents an enhanced diagram of electronic relaxations of CLN, taking into account presumably new emission bands in the band gap.

The analysis demonstrates that exact attribution of 1.54 and 1.36 eV bands is not quite clear, because the intensity of signal in SLN (6.0 wt% K_2_O) changes anomaly despite the fact that its composition is very close to stoichiometric one. The 1.54 and 1.36 eV luminescent bands can be additive, different luminescence center can overlap at these wavelengths. The number of luminescence centers will apparently depend on the donor and acceptor trapping levels, which are inextricably linked with Nb_Li_ defects. Possibly, there is a threshold value of Nb_Li_ defects; when it is approached from below, the luminescence in the near IR region decreases. The structure of the energy levels is rearranged after the threshold, the polaron luminescence is simultaneously enhanced. However, the kinetics of polaron luminescence excited by a pulsed source [53] shows monoexponential quenching of the 1.44 and 1.30 eV emission bands with the lifetimes τ_1_ = 0.42 μs and τ_2_ = 1.42 μs.

The SLN crystal was determined to have the least radiative recombination in the optical range due to the least amount of luminescent active defect centers. Luminescence centers with the participation of a Nb_Li_ defect make the main contribution to the luminescent signal of CLN crystal: Nb_Li_-Nb_Nb_ in the visible range, Nb_Li_-O in the near-IR range. SLN (6.0 wt% K_2_O) crystal has some residual amount of intrinsic Nb_Li_ defect that lead to an anomalous increase in the intensity of near-IR luminescence. The formation of a stable Nb_Li_-Nb_Nb_ bipolaron pair by radiative recombination is less probable than recombination involving the surrounding oxygens in the lithium octahedron. Therefore, the emission of the Nb_Li_-Nb_Nb_ ipolaron at ~2.04 eV in the volume PL in the visible region is weaker than the emission of the Nb_Li_O polaron in the near-IR region.

### 3.3. Thermal PL Quenching of CLN, SLN and SLN (6.0 wt% K_2_O) Crystals in the Visible and Near-IR Regions

**Figure 9:**
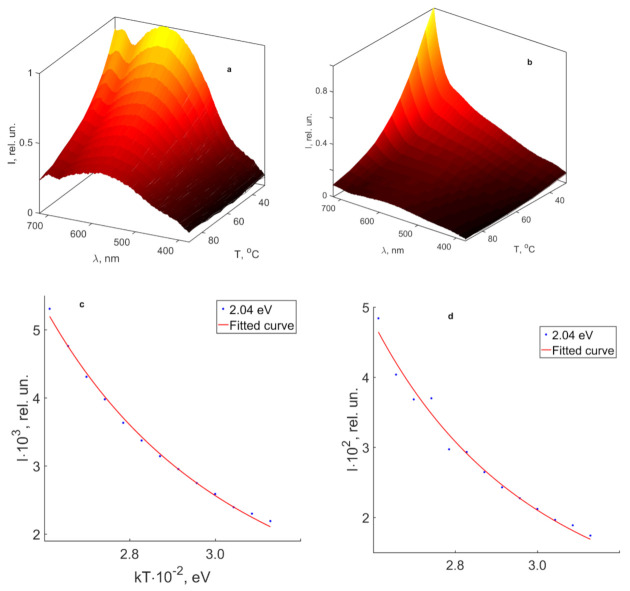
Temperature dependence of normalized PL intensity in the visible region and quenching of luminescence bands at 2.04 for crystals CLN (**a**,**c**) and SLN (6.0 wt% K_2_O) (**b**,**d**).

Figure 9 demonstrates changes in the luminescence intensity in the normalized PL spectra of CLN and SLN (6.0 wt% K_2_O) crystals in the temperature range 30–90 °C. As the crystal temperature increases, the amplitude of thermal vibrations of the crystal lattice (phonons) increases. The interaction of luminescence centers with lattice phonons increases the fraction of nonradiative processes, which is accompanied by a decrease in the luminescence intensity in the visible and near-IR regions. In addition, thermolysis of electrons from Nb_Li_^4+^ and Nb_Nb_^4+^ traps is possible, which also reduces the probability of radiative recombination and, consequently, the luminescence intensity. An increase in temperature quenches the PL of all crystals under study. Individual bands were distinguished from the spectrum as Gaussian curves due to data, obtained in Section 3.2. We obtained the activation energies of luminescence quenching for individual main peaks (1.36, 1.54 and 2.04 eV) by approximating the dependence I(T) by the Mott equation (Formula 1). The distinguishing was not carried out for SLN crystal in the visible region, since its luminescence in this area is too weak. Table 5 presents activation energies of various emission bands.

**Figure 10:**
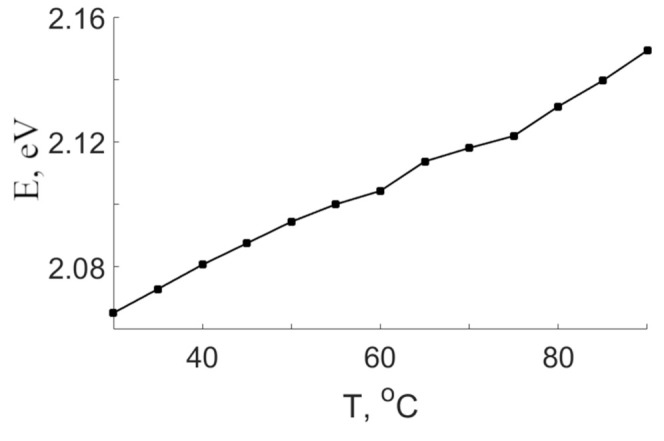
Dependence of the 2.04 eV maximum position on the CLN crystal temperature.

Energy activations for the 2.04 eV band of CLN and SLN (6.0 wt% K_2_O) crystals indicate similar nature of the luminescence center. The center is a bipolaron pair Nb_Li_-Nb_Nb_. The activation energy for this band in SLN (6.0 wt% K_2_O) crystal is E_a_ = 0.160 eV. This is 0.017 eV greater than E_a_ of the band in CLN crystal. The luminescence maximum at 2.04 eV shifts towards higher energies in the CLN crystal as the temperature increases, Figure 10. This indicates a shift in configuration coordinate of a bipolaron pair in this crystal. As the temperature increases, the amplitude of thermal vibrations of the lattice increases, which should lead to an increase in the average distance between the Nb atoms in its own and lithium site. In turn, an increased distance should lead to a decrease in the degree of overlap of d-d orbitals and, as a consequence, to a decrease in the probability of radiative recombination of the bipolaron. Radiative and nonradiative transitions of the bipolaron compete. The activation energy has a small value, 0.16 eV is sufficient to make nonradiative relaxations of the Nb_Li_-Nb_Nb_ pair a dominant mechanism with a slight increase in the crystal temperature. The latter explains the weak luminescence intensity in the visible region of all the crystals under study.

**Figure 11:**
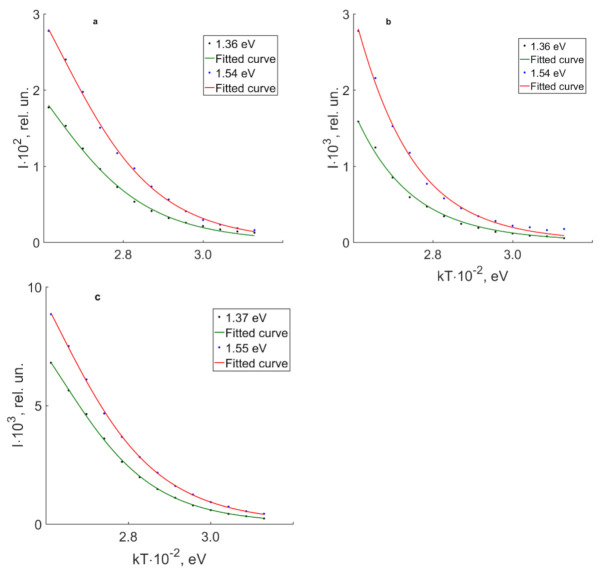
Curves of luminescence thermal quenching measured for 1.36 and 1.54 eV bands for crystals SLN (**a**), CLN (**b**), SLN (6.0 wt% K_2_O) (**c**).

Let us consider thermal quenching of near-IR luminescence maxima at 1.36 and 1.54 eV of nominally pure CLN, SLN and SLN (6.0 wt% K_2_O) crystals, Table 5. Figure 11 demonstrates dependencies of 1.36 and 1.54 eV luminescent bands intensities on temperature of crystals under study. Both bands have the smallest value of E_a_ for CLN crystal. This might indicate a ‘harder’ structure of SLN and SLN (6.0 wt% K_2_O) crystals; the oxygen octahedra are less distorted in them.

The obtained data reveal the high sensitivity of visible luminescence towards temperature. Even a small variation in the temperature quenches radiative recombination of Nb_Li_-Nb_Nb_ pair due to a decrease in the degree of overlap of d-d orbitals and increase in amplitude of lattice thermal vibrations. SLN and SLN (6.0 wt% K_2_O) crystals have a harder anion carcass than CLN.

## 4. Conclusions

Thus, the studied nominally pure SLN, CLN and SLN The (6.0 wt% K_2_O) crystals reveal a weak dependence of the surface luminescence at 2.75 eV on the composition of the crystal. This luminescence is due to intraconfigurational transitions in the NbO_6_ oxygen-octahedral cluster. Volume PL is caused by point defects of the cationic sublattice of studied samples. The maximum at 2.04 eV is due to the radiative recombination of Nb_Li_^4+^-Nb_Nb_^4+^ bipolaron pairs. A decrease in the distance between the niobium in the main position (Nb_Nb_) and the Nb_Li_ point defect increases the fraction of overlapping d–d orbitals and, as a consequence, the emission probability at 2.04 eV increases. Bipolaron luminescence is maximal in CLN. Bipolaron luminescence in SLN is quenched because there are a few bipolarons in the crystal and small probability of their radiative recombination. At the same time, SLN (6.0 wt% K_2_O) crystal does have Nb_Li_^4+^-Nb_Nb_^4+^ luminescent centers; the fact is caused by a small amount of Nb_Li_ defects.

Polaron luminescence in the near-IR region depends on the crystal stoichiometry. 1.54 eV band is caused by Nb_Li_^4+^-O^−^ pair, 1.34 eV—by a transition between Nb_Nb_^4+^ and Nb_Li_^5+^. The difference corresponds to 2.85–1.54 = 1.31 eV, which correlates well with the position of the last maximum. The SLN crystal has the lowest radiative recombination in the optical range due to a smaller number of luminescent active defect centers. Luminescence centers with the participation of the Nb_Li_ defect make the main contribution to the CLN luminescent signal in the visible and near-IR regions. The defects are Nb_Li_^4+^-Nb_Nb_^4+^ bipolaron pairs and Nb_Li_-O defect. SLN (6.0 wt% K_2_O) crystals have a residual number of intrinsic point defects Nb_Li_; part of them lead to an anomalous increase in the intensity of the luminescence in the near-IR region. This indicates the additive nature of the mechanisms of radiative recombination with the formation of “new” luminescence centers different from the centers with the participation of Nb_Li_ defects. PL in visible and near-IR is thermally quenched. Thermal quenching of the Nb_Li_-Nb_Nb_ bipolaron pair occurs due to a decrease in the overlap of d–d orbitals due to an increase in the amplitude of thermal vibrations of the lattice. In this case, the SLN and SLN (6.0 wt% K_2_O) crystals have a “harder” anionic lattice framework than the CLN crystal.

## Figures and Tables

**Table 1:** Optical characteristics of the crystals, Δλ_abs_ = ±0.1 nm.

Crystal	λ_abs_, nm	R	E_U,_ meV	E_g1_, eV	E_g2_, eV	E_p_, cm^−1^	Phonons
**CLN**	330.0	0.948	94.4	3.813	3.644	114	120 cm^−1^
**SLN**	326.9	0.952	93.2	3.846	3.540	1187	complex phonons
**SLN(6.0 wt% K_2_O)**	326.0	-	50.7	3.826	3.690	328	323 or 333 cm^−1^

**Table 2:** Spectral characteristics of separated surface luminescence bands of the studied samples.

The Band		1	2	3	4	5	6	7
CLN	I, rel. un.	0.008	0.005	0.064	0.086	0.142	0.099	0.004
	E, eV	2.144	2.268	2.491	2.636	2.776	2.914	3.046
	ΔE, eV	0.296	0.141	0.255	0.174	0.153	0.132	0.111
SLN	I, rel. un.	0.010	0.025	0.059	0.098	0.133	0.124	0.007
	E, eV	2.064	2.322	2.518	2.661	2.796	2.940	3.088
	ΔE, eV	0.286	0.284	0.231	0.186	0.153	0.154	0.129
SLN (6.0 wt% K_2_O)	I, rel. un.	0.014	0.050	0.066	0.094	0.143	0.115	0.012
	E, eV	2.064	2.315	2.519	2.663	2.805	2.952	3.095
	ΔE, eV	0.268	0.308	0.229	0.176	0.155	0.145	0.145

**Table 3:** Spectral characteristics of individual volume PL bands of the crystals under study.

Luminescence Band		1	2	3	4
CLN	I, rel. un.	2321	-	28,105	3464
	E, eV	1.767	-	2.035	2.846
	ΔE, eV	0.055	-	0.841	0.710
SLN	I, rel. un.	3609	954	4175	1040
	E, eV	1.696	1.941	2.061	2.899
	ΔE, eV	0.200	0.205	0.893	0.686
SLN (6.0 wt% K_2_O)	I, rel. un.	1244	5393	14,677	4547
	E, eV	1.780	1.886	2.043	2.869
	ΔE, eV	0.134	0.230	0.779	0.850

**Table 4:** Spectral characteristics of individual bands after the PL spectra decomposition of CLN, SLN and SLN (6.0 wt% K_2_O) crystals in the near-IR region.

Luminescence Band		1	2	3	4	5	6	7
CLN	I, rel. un.	585	994	9501	851	18,647	1175	-
	E, eV	1.243	1.346	1.360	1.455	1.540	1.638	-
	ΔE, eV	0.024	0.029	0.237	0.054	0.291	0.045	-
SLN	I, rel. un.	693	538	8448	845	14,621	1363	269
	E, eV	1.242	1.347	1.359	1.454	1.530	1.637	1.696
	ΔE, eV	0.021	0.044	0.226	0.059	0.253	0.065	0.019
SLN (6.0 wt% K_2_O)	I, rel. un.	622	2662	34,680	2679	43,711	4931	1042
	E, eV	1.238	1.345	1.374	1.449	1.545	1.634	1.694
	ΔE, eV	0.036	0.037	0.235	0.089	0.225	0.069	0.022

**Table 5:** The position of the maxima and the value of the activation energy for the crystals under study.

Crystal	E_1_, eV	E_a_, eV	E_2_, eV	E_a_, eV	E_3_, eV	E_a_, eV
CLN	2.04	0.143	1.36	0.541	1.54	0.584
SLN	-	-	1.36	0.599	1.53	0.618
SLN (6.0 wt% K_2_O)	2.04	0.160	1.37	0.672	1.55	0.620

## Data Availability

The raw data supporting this study is available from a corresponding author, D.M., on a reasonable request.
